# Transcription of the Major *Neurospora crassa* microRNA–Like Small RNAs Relies on RNA Polymerase III

**DOI:** 10.1371/journal.pgen.1003227

**Published:** 2013-01-17

**Authors:** Qiuying Yang, Liande Li, Zhihong Xue, Qiaohong Ye, Lin Zhang, Shaojie Li, Yi Liu

**Affiliations:** 1 Department of Physiology, The University of Texas Southwestern Medical Center, Dallas, Texas, United States of America; 2 School of Life Sciences, Sun Yat-sen University, Guangzhou, China; 3 State Key Laboratory of Mycology, Institute of Microbiology, Chinese Academy of Sciences, Beijing, China; Oregon State University, United States of America

## Abstract

Most plant and animal microRNAs (miRNAs) are transcribed by RNA polymerase II. We previously discovered miRNA–like small RNAs (milRNAs) in the filamentous fungus *Neurospora crassa* and uncovered at least four different pathways for milRNA production. To understand the evolutionary origin of milRNAs, we determined the roles of polymerases II and III (Pol II and Pol III) in milRNA transcription. Our results show that Pol III is responsible for the transcription of the major milRNAs produced in this organism. The inhibition of Pol III activity by an inhibitor or by gene silencing abolishes the production of most abundant milRNAs and pri–milRNAs. In addition, Pol III associates with these milRNA producing loci. Even though silencing of Pol II does not affect the synthesis of the most abundant milRNAs, Pol II or both Pol II and Pol III are associated with some milRNA–producing loci, suggesting a regulatory interaction between the two polymerases for some milRNA transcription. Furthermore, we show that one of the Pol III–transcribed milRNAs is derived from a tRNA precursor, and its biogenesis requires RNase Z, which cleaves the tRNA moiety to generate pre–milRNA. Our study identifies the transcriptional machinery responsible for the synthesis of fungal milRNAs and sheds light on the evolutionary origin of eukaryotic small RNAs.

## Author Summary

microRNAs (miRNAs) are small RNAs that are used by many organisms to regulate a wide variety of molecular, developmental, and physiological activities. In higher eukaryotes, such as animals and plants, the majority of the independent transcribed miRNAs are produced by RNA polymerase II (Pol II), an enzyme that is also responsible for the production of most of the messenger RNAs. On the other hand, only a few tRNA–associated miRNAs are known to be produced by RNA polymerase III (Pol III), an enzyme that is responsible for the production of small sized RNAs such as tRNAs and 5s rRNA. We previously identified the first fungal miRNAs by identifying the small RNAs associated with an Argonaute protein in the filamentous fungus *Neurospora crassa*. In this study, we examined the role of Pol II and Pol III in the production of *Neurospora* miRNAs. We showed that, unlike in plants and animals, Pol III appears to be a major RNA polymerase responsible for miRNA production in this fungus. Our study identified the transcriptional machinery responsible for the synthesis of fungal miRNAs and shed light on the evolutionary origin of miRNAs.

## Introduction

MicroRNAs (miRNAs) are small non-coding RNAs that regulate protein expression levels post-transcriptionally in a wide variety of cellular processes. miRNAs arise from discrete genomic loci and are processed by Dicer-like enzymes from stem-loop RNA precursors [1], [2]. Since their initial discovery in *Caenorhabditis elegans*, miRNAs have been found in animals, plants and algae [3]–[8]. Most animal and plant miRNAs are transcribed by RNA polymerase II (Pol II) [9]–[12]. Pol II-mediated transcription allows spatial and temporal regulation of the expression of miRNAs. In eukaryotic organisms, RNA polymerase III (Pol III) is the polymerase responsible for the transcription of tRNAs, 5S ribosomal RNA and some small nuclear RNAs, which are all short non-coding RNAs [13], [14].

Compared to transcriptional regulation of Pol II, the regulation of Pol III-mediated transcription is less complex, thus its role in transcribing housekeeping RNAs. Despite their different transcriptomes, Pol II and Pol III are evolutionarily related; the two, share some catalytic subunits, and many of their specific subunits are homologous to each other. In addition, the polymerases share transcription factors and promoter elements to a certain degree, which allows crosstalk between the two enzymes [15]–[17]. Recently, a small number of mammalian and viral miRNAs have been shown to be transcribed by Pol III [12], [18]–[21]. Most of these miRNAs derive from tRNA precursors or are transcribed from regions with repetitive elements.

We previously discovered the first fungal miRNA-like small RNAs (milRNAs) in the filamentous fungus *Neurospora crassa* [22]. Like plant and animal miRNAs, the *Neurospora* milRNAs arise from transcripts with stem-loop structures and are mostly dependent on Dicer for production. In addition, the vast majority of milRNA sequences correspond to one arm of the hairpin of the precursor (the milRNA arm) and have a strong preference for U at the 5′end. Interestingly, there are at least four different milRNA production mechanisms that use distinct combinations of factors, including Dicers, QDE-2 and the exonuclease QIP [23]. Studies of the *Neurospora* milRNAs shed light on the diversity and evolutionary origins of eukaryotic miRNAs.

In this study, we examined the role of Pol II and Pol III in milRNA biogenesis. Our results showed that Pol III is responsible for most milRNAs production in this fungus. On the other hand, Pol II was found to be associated with some *milR* loci, suggesting a collaboration between the two polymerases in milRNAs production. Furthermore, *milR-4* milRNA was derived from a tRNA precursor and requires RNase Z for production.

## Results

### milRNA production reduced by a Pol III–specific inhibitor

We previously identified ∼25 putative *milR* loci by analyzing the QDE-2-associated small RNAs [22]. Among these *milRs*, *milR-1, -2, -3* and *-4* were experimentally confirmed due to their relative high expression levels. For other miRNA genes, even though they were identified by deep sequencing the QDE-2 associated small RNAs, they could not be detected by Northern blot analysis due to their low abundance. It is worth noting that the *milR-1, -2, -3* and *-4* genes account for the vast majority (92%) of all milRNAs produced in *Neurospora*. Therefore, we mostly focused our analyses on these four major milRNAs.

**Figure 1:**
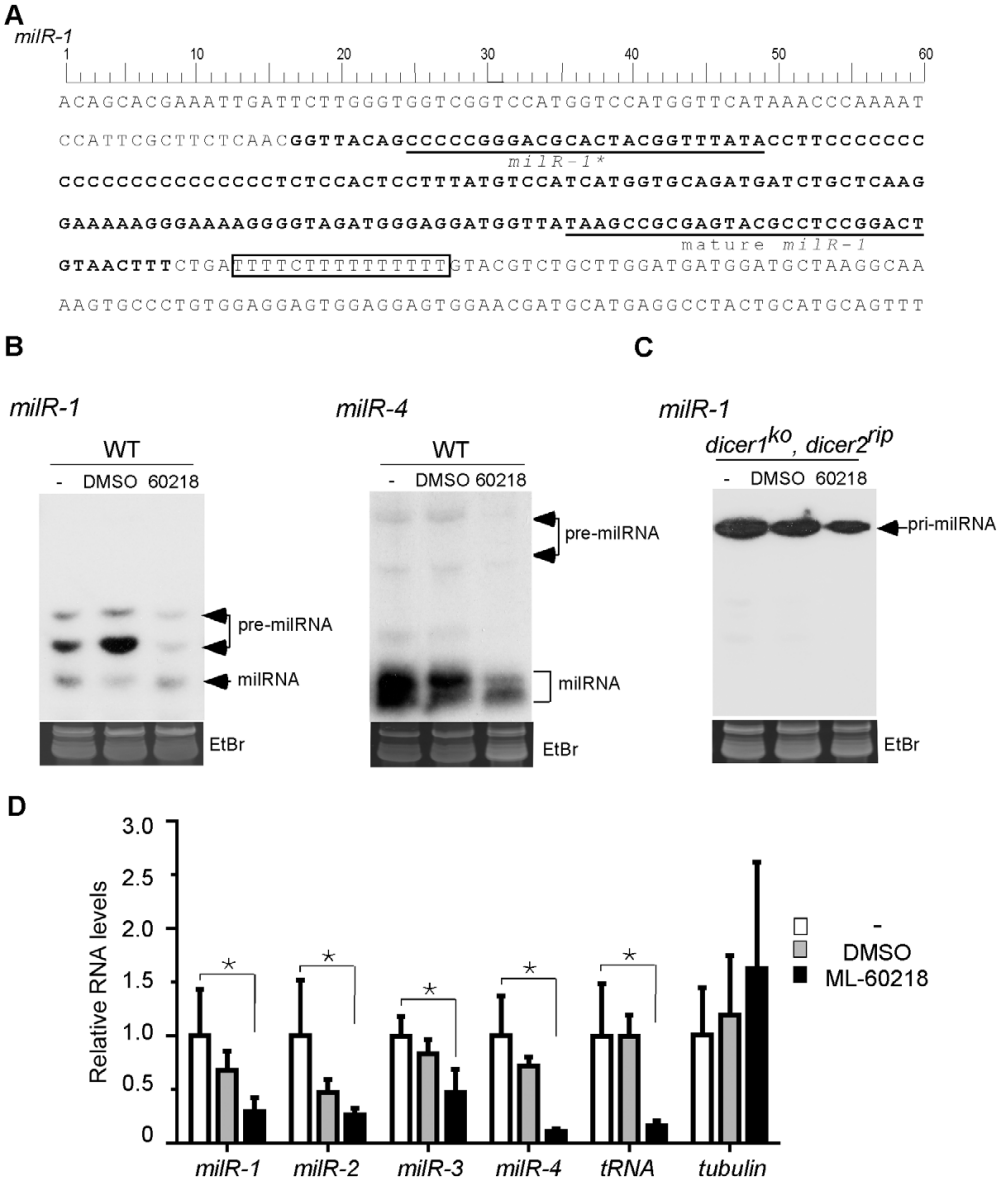
Pol III is required for the production of milRs. A. DNA sequence of *milR-1* locus. B. Northern blot showing the level of small RNAs in indicated strains after indicated treatments. ML-60218 is Pol III specific inhibitor and DMSO is used as the control. The cultures were treated with ML-60218 before they were collected. The ethidium bromide-stained gel in the bottom panel shows equal loading of RNA samples. C. Northern blot result showing the levels of *pri-milR-1* in *dicer* double knockout strain in the presence of the inhibitor. D. qRT-PCR analyses showing that the production of pri-milRNAs for *milR-1*, *milR-2*, *milR-3* and *milR-4* was inhibited by ML-60218. rRNA level was used as the loading control in the qRT-PCR analysis. *β-tubulin* and a Pol III-transcribed tRNA was served as the negative and positive control, respectively. WT indicates the wild-type strain. The asterisks indicate *P* value<0.05. Error bars indicate S.D.

**Figure 2:**
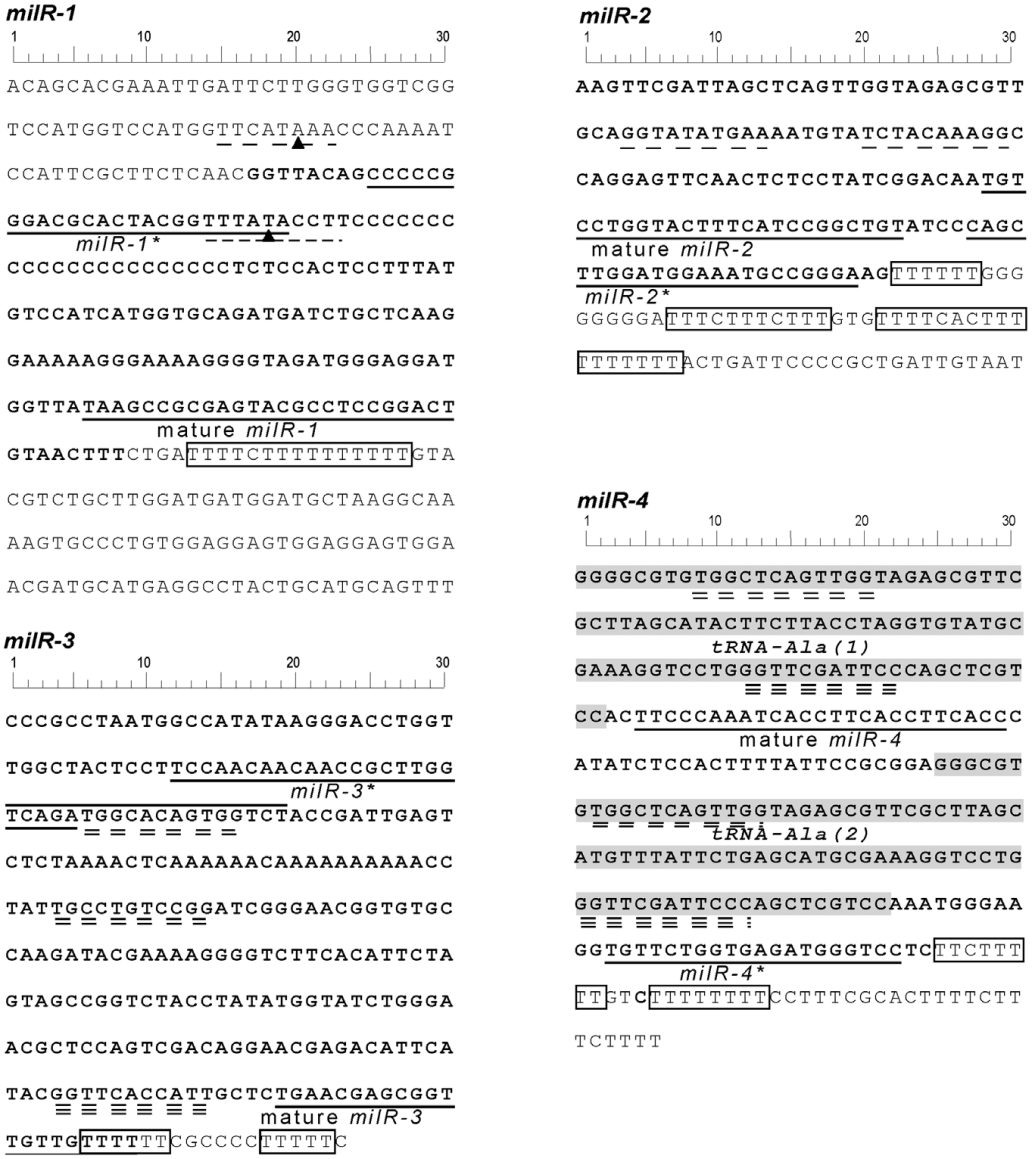
DNA sequences of *milR-1-4*. The sequences of milRNA, milRNA* and the poly T sequences are indicated. TATA-like elements are underlined with single dashed lines. Putative A-boxes and B-boxes are underlined with double and triple dashed lines, respectively. The solid triangles in the *milR-1* sequence indicate the nucleotides mutated in our promoter analysis.

*milR-1* is the most abundant milRNA-producing locus in the *Neurospora* genome. It is localized in the intergenic region between two predicted *Neurospora* genes [22]. The primary-milRNA (pri-milRNA) transcripts produced from *milR-1* are ∼170 nucleotides (nt), much shorter than most of the known Pol II transcripts in *Neurospora*. Sequence analyses of *milR-1* and other experimentally confirmed milR genes (*milR-2*, *milR-3*, and *mil-4*) that produce abundant milRNAs revealed that these four all share a stretch of T residues immediately downstream of the milRNA sequences (Figure 1A and Figure 2). The T cluster is a signature sequence motif for the transcription terminator of Pol III [13], [24], raising the possibility that the pri-milRNAs that generate these milRs are transcribed by Pol III.

To examine the involvement of Pol III in milRNA production, we treated *Neurospora* cultures with ML-60218, a Pol III specific inhibitor [25]. Small RNA analysis by northern blot showed that the levels of both forms of *pre-milR-1* were dramatically reduced in the presence of the inhibitor (Figure 1B). Similarly, ML-60218 also inhibited the production of *pre-milR-4* and mature *milR-4*. To determine whether the inhibition of *milR-1* production is due to the inhibition of the *pri-milR-1* production, we examined the levels of *pri-milR-1* in a *dicer* double mutant, in which the pri-milRNAs accumulate to a high level. As shown in Figure 1C, ML-60218 also inhibited the production of *pri-milR-1*. Quantitative RT-PCR analyses showed that levels of tRNA, *pri*-*milRNA-1, -2, -3* and -*4* were reduced in the presence of the inhibitor in the wild-type strain (Figure 1D). In contrast, the levels of *β-tubulin* transcript were not affected by treatment of cultures with the inhibitor, indicating specific inhibition of Pol III. These results suggest that these milRNAs might be transcribed by Pol III.

### Pol III is required for transcription of *milR-1-4* and associates with many *milR* loci

**Figure 3:**
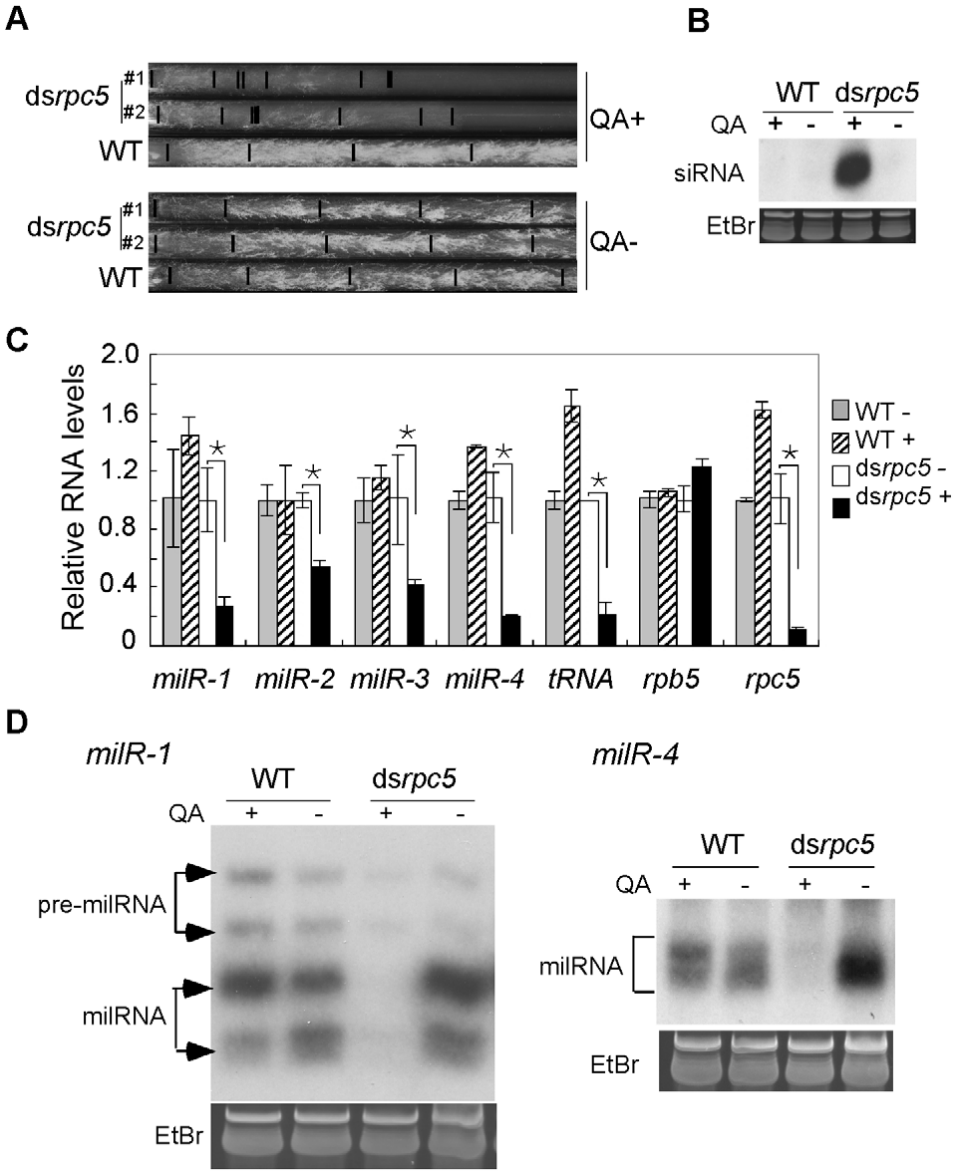
Pol III knockdown results in the reduction of milRNA expression. A. Race tube assays showing the growth rates of the indicated strains in the presence or absence of QA. B. Northern blot analysis showing the levels of *rpc5*-specific siRNA in the indicated strains. C. qRT-PCR analysis showing the reduction of pri-milR levels when *rpc5* was silenced. rRNA level was used as the loading control for qRT-PCR. A Pol III-transcribed tRNA was served as a positive control. D. Northern blot showing the levels of *milR-1* and *milR-4* small RNAs.

*rpc5* is an essential *Neurospora* gene that encodes the *Neurospora* homolog of RPC34, a Pol III-specific subunit in *Saccharomyces cerevisiae*. To obtain genetic evidence for the involvement of Pol III in milR transcription, we generated *Neurospora* strains (ds*rpc5*) in which the expression of *rpc5* (NCU00727) can be inducibly silenced by an *rpc5*-specific dsRNA in the presence of quinic acid (QA) inducer. In the absence of QA, race tube assays showed that the ds*rpc5* strain grew similarly to the wild-type strain (Figure 3A). The addition of QA to the growth media, however, resulted in a dramatic reduction of growth rate of the ds*rpc5* strain and the induction of *rpc5*-specific siRNA (Figure 3A and 3B), indicating the specific silencing of *rpc5* expression.

qRT-PCR analyses showed that the silencing of *rpc5* by QA in the ds*rpc5* strain resulted in significant reduction of pri-milRNA levels for the four experimentally confirmed milRs that produce vast majority of milRNAs in *Neurospora* (Figure 3C). In contrast, QA had no inhibitory effects on the pri-milRNA levels in the wild-type strain. Northern blot analyses showed that, as expected, the levels of mature *milR*-*1* and *milR-4* were also reduced when *rpc5* was silenced in the ds*rpc5* strain (Figure 3D). These data indicate that Pol III is required for the transcription of these milRNAs.

**Figure 4:**
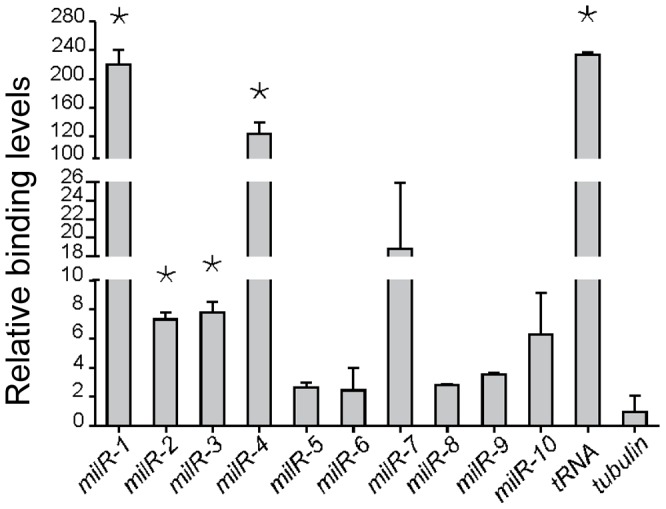
Pol III specifically binds to milR loci. ChIP assay results showing the binding of Pol III to the milR loci. A *tRNA* and the *β-tubulin* gene was used as the negative and positive control, respectively. The asterisk indicate *P* value<0.05. Error bars indicate S.D.

To examine whether the involvement of Pol III in milR transcription is direct, we created a *Neurospora* strain in which a Pol III-specific subunit, RPC7 (NCU07656), is c-Myc tagged, and performed chromatin immunoprecipitation (ChIP) assays using a monoclonal c-Myc antibody for ten of the milRNA-producing loci (*milR-1* - *milR-10*). As shown in Figure 4, a strong RPC7-DNA binding was detected at a selected tRNA locus. In contrast, only background DNA binding was detected at the *β-tubulin* locus, indicating the specificity for Pol III-DNA binding in our ChIP assays. Significant RPC7-DNA binding by Pol III was detected in the *milR-1*, *milR-2*, *milR-3* and *milR-4* loci. For *milR-7* and *milR-10* loci, moderate binding was observed. For *milR-5*, *milR-6*, *milR-8* and *milR-9* loci, however, slightly higher than background binding was observed. These results suggest that Pol III is directly involved in the transcription of many of the *milRs* in *Neurospora*.

### Pol II is involved in the transcription of a set of less abundant *milRs*

**Figure 5:**
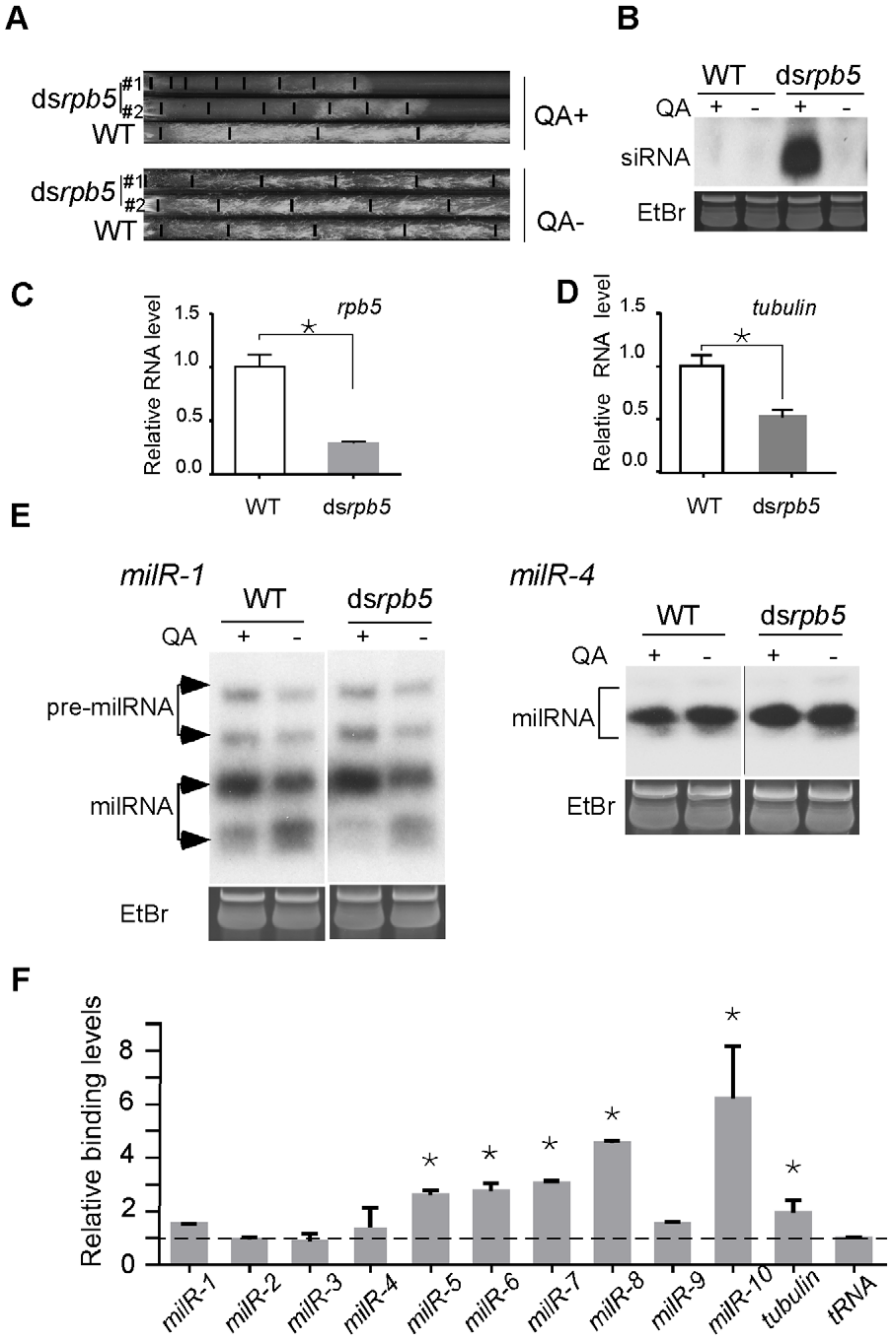
The involvement of Pol II in milRNA production. A. Race tube assays showing the growth rates of the indicated strains in the presence or absence of QA. B. Northern blot analysis showing the levels of *rpb5*-specific siRNA in the indicated strains. C and D. qRT-PCR analysis results showing the reduction of *rpb5 and β-tubulin* level in ds*rpb5* strain in the presence of QA. The asterisks indicate *P* value<0.05. Error bars indicate S.D. E. Northern blot analysis showing the levels of *milR-1* and *milR-4* small RNAs. F. ChIP assay using c-Myc antibody showing the binding of Pol II to milR loci in the Myc-RPB6 strain. A *tRNA* and the *β-tubulin* gene was served as the negative control and positive control, respectively.

The vast majority of animal and plant miRNAs are transcribed by Pol II. To determine the involvement of Pol II in milRNA production in *Neurospora*, we created a ds*rpb5* strain in which *rpb5* (NCU02661), the gene encoding for a subunit of Pol II homologous to RPB7 in yeast [26], can be inducibly silenced by addition of QA to the culture media. As shown in Figure 5A and 5B, the addition of QA resulted in significant growth inhibition and the production of *rpb5*-specific siRNA in the ds*rpb5* strain but not in the wild-type strain, indicating the specific silencing of the Pol II subunit in the ds*rpb5* strain. As shown in Figure 5C and 5D, the reduced levels of *rpb5* and *β-tubulin* in the presence of QA suggest the silencing of pol II-mediated transcriptin. As shown in Figure 5E, the silencing of *rpb5* had no significant effects on the production of the mature milRNAs from the *milR-1* and *milR-4* genes. Similar results were also obtained by silencing another Pol II-specific subunit RPB6 NCU09378 (data not shown). These results suggest that Pol II is not required for the transcription of these abundant milRs.

The observed weak binding between the Pol III-specific subunit RPC7 and several of the milR loci in ChIP assays raises the possibility that Pol II may be involved in the transcription of these low abundance milRs. To test this, we created a Myc-tagged RPB6 strain and performed ChIP assays using the c-Myc antibody to examine the association of Pol II with these *milR* loci. For *milR-5*, *milR-6* and *milR-8* loci, significant binding was observed, suggesting that Pol II is involved in transcription of these milRNAs. Interestingly, high levels of RPB6-DNA binding were also observed in the *milR-7* and *milR-10* loci, where Pol III also associates (Figure 5F). In contrast, only background RPB6-DNA binding was observed at the *milR-1*, *milR-2*, *milR-3* and *milR-4* loci that produce the vast majority of milRNAs, further supporting the hypothesis that the transcription of these highly abundant milRNAs is solely mediated by Pol III. These results suggest that even though Pol III plays a major role in transcribing the abundant milRs, Pol II is also involved in the transcription of some of milRs.

**Figure 6:**
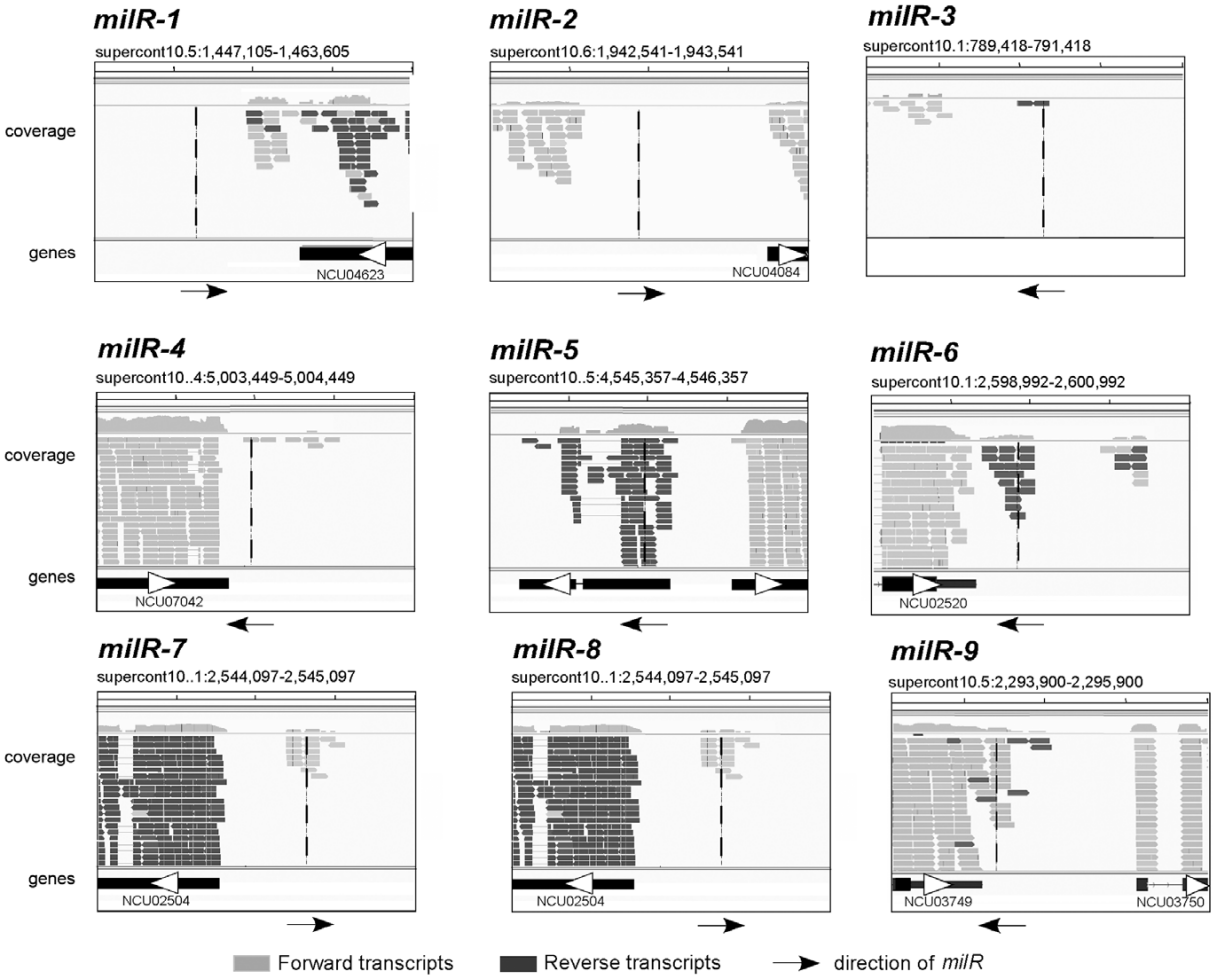
RNA sequencing of poly(A) RNA results showing the presence/absence of Pol II transcripts in the selected milR loci. Viewing window was set as 2000 nt. The vertical line in each panel indicates the location of the indicated *milR* gene.

To examine the involvement of pol III in the transcription of these low abundance milRs, we performed RNA sequencing using poly(A)-containing RNA of a wild-type strain. As shown in Figure 6, even though *milR-1*, *-2*, *-3* and *-4* loci are responsible for 92% of all *Neurospora* milRNAs, there was no or only background RNA reads, further supporting the conclusion that they are transcribed by Pol III. On the other hand, poly(A)-containing RNA reads were seen in the *milR-5*, *-6*, *-7*, *-8* and *-9* loci, suggesting that Pol II is involved in the transcription of these low abundance milRs.

### *milR-1's* transcription is mediated by a non-conventional Pol III promoter

**Figure 7:**
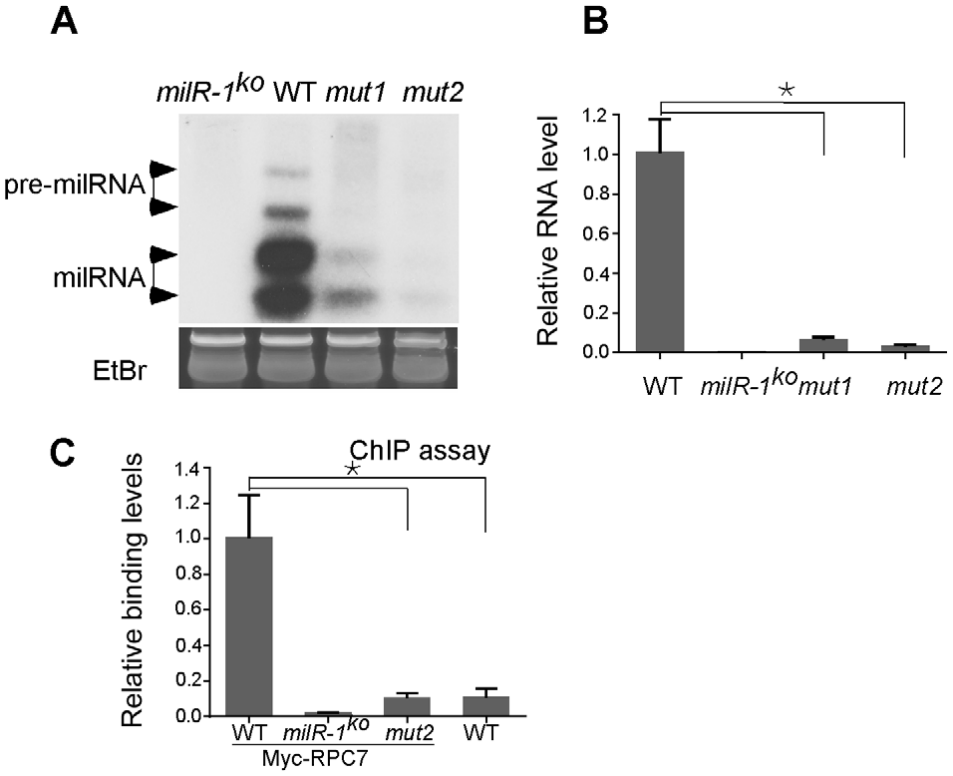
*milR-1* transcription is mediated by a non-conventional Pol III promoter. A. Northern blot analysis showing the levels of *milR-1* in the indicated strains. B. qRT-PCR analysis showing the reduction of *pri-milR-1* levels in the *mut1* and *mut2* strains. WT and *milR-1^ko^* served as the positive and negative control, respectively. C. ChIP assays using the c-Myc antibody showing the reduced binding of Myc-RPC7 at the *milR-1* locus with the mutated TATA-like element. The asterisk indicates *P* value<0.05. Error bars indicate S.D.

The *milR-1* locus has no predicted Pol III-specific A box or B box promoter elements [13], [27]. Instead, two putative TATA-like elements reside both inside and outside of the *pri-milR-1* region (Figure 2). It is known that Pol III can recognize TATA elements for transcription initiation of some genes [13]. To examine the role of these elements in *milR-1* transcription, two *milR-1* mutant constructs, (*mut1* and *mut2*), in which one of the TATA-like elements was mutated by point mutations, were created and transformed into a *milR-1* knock-out strain. Northern blot analysis showed that *milR-1* production was dramatically reduced in the *mut1* and *mut2* strains (Figure 7A). qRT-PCR analysis further showed that the *pri-milR-1* levels were also dramatically reduced in the mutants (Figure 7B), suggesting that both of these elements are important for the *milR-1* transcription. To determine the role of these elements in Pol III recruitment, the Myc-RPC7 construct was transformed into the wild-type, *milR-1^KO^* and *mut2* strains. ChIP assay using the c-Myc antibody was performed to compare the binding of Myc-RPC7 at the *milR-1* locus in these strains. A wild-type strain without the Myc-RPC7 construct was used as a control. As shown in Figure 7C, the RPC7 binding at the *milR-1* locus was dramatically reduced in the *mut2* strain, indicating that this TATA-like element is essential for the recruitment of Pol III to *milR-1*. These results suggest that *milR-1* uses a non-conventional Pol III promoter for its transcription.

### *milR-4* derives from a tRNA–containing transcript and requires RNase Z for production

**Figure 8:**
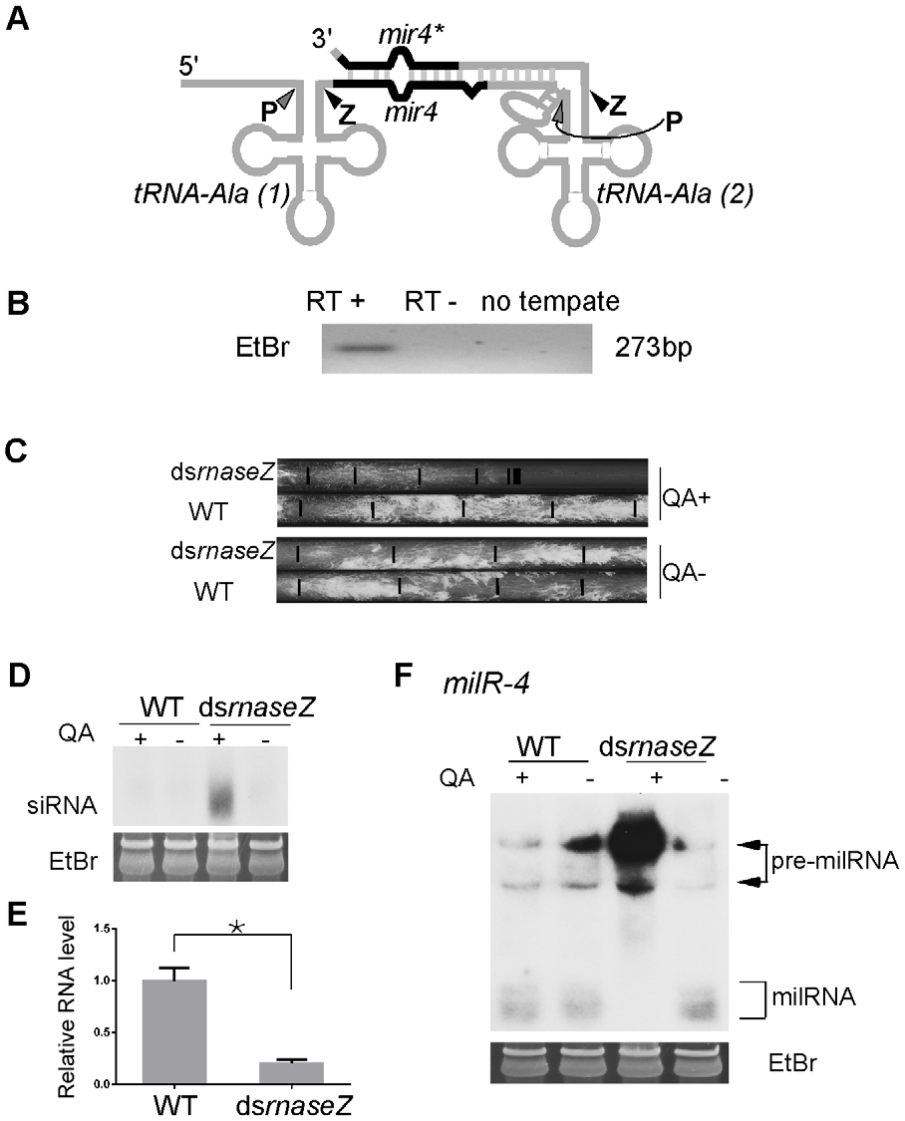
RNase Z is required for *milR-4* processing. A. A Diagram showing the predicted secondary structure of *pri-milR-4*. B. RT-PCR analysis, which used a pair of primers indicated in A, showing the presence of transcript that spanning the two alanine tRNAs and *milR-4*. C. Race tube assays showing the growth rates of the indicated strains in the presence or absence of QA. D. Northern blot analysis showing the levels of *rnaseZ*-specific siRNA in the indicated strains. E. qRT-PCR analysis showing the reduction of *rnaseZ* mRNA level in the ds*rnaseZ* strain in the presence of QA. The asterisk indicates *P* value<0.05. Error bars indicate S.D. F. Northern blot analysis showing the levels of *milR-4* milRNAs in the indicated strains in the presence or absence of QA.

In eukaryotic organisms, Pol III is the RNA polymerase that transcribes tRNA, 5S ribosomal RNA and other small nuclear RNAs. The major role of Pol III in milR transcription suggests an evolutionary link between milRNAs and these small non-coding RNAs. Although most milRs are found in intergenic regions and are not adjacent to these small RNA genes, the mature *milR-4* is located between the predicted genes for two alanine tRNAs and the milRNA* strand is downstream of the second tRNA (Figure 8A), suggesting that *milR-4* may derive from RNA transcripts that contain these two tRNAs. To examine this, we performed RT-PCR analysis using a pair of primers that flank the two tRNA^Ala^ regions. As shown in Figure 8B, an RT-PCR specific fragment of the predicted size was observed, indicating that *milR-4* is indeed co-transcribed with the two tRNAs.

After the transcription by Pol III, eukaryotic tRNA precursors are processed at the 5′ and 3′ ends to remove the extraneous sequences, resulting in the generation of functional tRNAs [28]–[30]. The 3′ end sequences of pre-tRNAs are cleaved by the endonuclease RNase Z or other nucleases to generate mature 3′ends of tRNAs. We have previously shown that the production of *milR-4* milRNA is partially dependent on Dicer [22]. The predicted RNA secondary structure of *tRNA-milR-4* precursor suggests that Dicer should cut the stem region at the 3′ end of milRNA precursor and that another nuclease generates the 5′ end (Figure 8A). Because of predicted processing by RNase Z downstream of the first tRNA, we reasoned that RNase Z is required for the maturation of *milR-4*. Because RNase Z is likely essential for *Neurospor*a cell growth, we created a strain (ds*rnaseZ*) in which the expression of the gene that encodes the *Neurospora* homolog of RNase Z (NCU00232) can be inducibly silenced by dsRNA. The addition of QA resulted in the production of *rnaseZ*-specific siRNA and dramatic inhibition of cell growth in the race tube assay in the ds*rnaseZ* strain but not in the wild-type strain (Figure 8C and Figure 8D). qRT-PCR further showed that *rnaseZ* level was down-regulated in the presence of QA (Figure 8E). In contrast, the growth rates of both strains were comparable in the absence of QA, indicating that the silencing of RNase Z is inducible and specific. As shown by the northern blot analysis result in Figure 8F, the silencing of *rnaseZ* expression by QA almost completely abolished the production of mature milRNA and resulted in the accumulation of high molecular weight pre-milRNA species. This result indicates that the nuclease RNase Z is required for the maturation of *milR-4*; it processes the 3′ end of the upstream tRNA^Ala^ in the transcript containing *milR-4* to generate the 5′ end of the milRNAs.

## Discussion

Our results demonstrated that RNA polymerase II and III are both involved in the transcription of the milRNAs in *Neurospora*. Since *milR-1-4* account for the vast majority of milRNAs in *Neurospora* [22], Pol III is a major RNA polymerase for miRNA production in this organism. We showed that both the treatment of *Neurospora* by a Pol III-specific inhibitor and the silencing of a Pol III subunit by RNAi resulted in the near abolishment of the production of *milR-1* and *milR-4*. In addition, the levels of pri-miRNAs of *milR-1*, *-2*, *-3* and -*4* were also reduced upon inhibition of Pol III. Furthermore, we showed that a Pol III-specific subunit is specifically associated with milR loci *1*, *2*, *3* and *4*, indicating that Pol III is directly involved in mediating milR transcription.

The prominent role of Pol III in *Neurospora* milRNA transcription is in contrast with its minor role in miRNA production in animals and plants [9]–[12]. It was recently shown that Pol III is responsible for the transcription of a few animal miRNAs, mostly those derived from tRNA precursors and regions of repetitive elements [12], [18]–[20], [31]. Even though most of the *Neurospora* milR genes are located in intergenic regions with no repetitive elements, we showed that *milR-4*, one of the most abundant *Neurospora* milRNAs, is derived from a region that also encodes tRNAs. Moreover, the maturation of *milR-4* requires RNase Z. Our results suggest that RNase Z cleaves the pre-tRNA to generate the 5′ end of *pre-milR-4*. It is likely that the *pre-milR-4* is further processed by Dicer. The production mechanism of *milR-4*, therefore, is very similar to the biogenesis pathway of the murine γ-herpesvirus 68 encoded miRNAs, which are also cleaved by RNase Z from a tRNA moiety to generate pre-miRNA [19]. Therefore, our results indicate that the tRNA and RNase Z-mediated miRNA production mechanism is conserved from fungi to mammals.

Despite the lack of a significant role for Pol II in the transcription of the most abundant milRNAs, our ChIP assays showed that Pol II was associated with several milR loci at which Pol III binding is low. Poly (A) RNA sequencing results suggest that Pol II transcription is involved in the production of these low abundance milRNAs. Also interestingly, specific Pol III binding was also observed at the *milR-7* locus, which also has poly (A) transcripts, suggesting that both polymerases may contribute to the transcription of these milRNAs. It has been previously suggested that these Pol II and III can cooperate to enhance the transcription in mammalian cells [16], [32].

miRNAs function in a wide variety of cellular and developmental processes in animal and plants [1], [33]. The use of Pol II allows the expression of miRNAs to be spatially and temporally regulated. Pol III is normally used for the transcription of small housekeeping non-coding RNAs, including tRNAs, 5S ribosomal RNA and snRNAs. Although the *Neurospora* milRNAs, like animal miRNAs, can silence endogenous targets with mismatches *in vivo*, their physiological functions are still unclear [22]. The fact that most *Neurospora* milRNAs are transcribed by Pol III suggests that expression of these *Neurospora* small RNAs is not as highly regulated as the production of animal and plant miRNAs. On the other hand, the binding of Pol II alone and the binding of both Pol II and Pol III to some of the milR loci suggest that the transcription of milRNAs may be flexible. Such flexibility may be explained by the fact that both polymerases share some of the same subunits and can recognize similar promoter elements, which allows regulatory interactions between the two enzymes [14]. Alternatively, it is possible that when fungal milRNAs first evolved, they were initially Pol III products. When a milRNA adopted a specific function that had to be highly regulated, interactions between Pol II and Pol III may have resulted in its transcription by Pol II. Future experiments that reveal the functions of different fungal milRNAs should shed light on the biogenesis and evolutionary origins of eukaryotic miRNAs.

## Materials and Methods

### Strains and growth conditions

The wild-type strain used in this study is FGSC 4200(a). The *his-3* locus targeting transformation vector qa.pDE3dBH was used to construct double strand RNA knockdown and Myc-tagged expression vectors. The QA-inducible knockdown strains for ds*rpc5*, ds*rpb5*, ds*rnaseZ* and the QA-inducible Myc-tagged expression strains harboring qaMyc-RPB6 and qaMyc-RPC7 were introduced into the *his-3* locus of a wild-type strain (301-6, *his-3*) as described previously [34]. *milR-1^ko^* was generated by gene replacement to delete the entire *milR-1* locus with the hygromycin resistance gene (*hph*), as previously described [35]. Single nucleotide mutation for *milR-1* promoter mutants, *mut1* and *mut2*, was performed using one-step site-directed and site-saturation mutagenesis method [36]. The mutation vector was also transformed into *Neurospora* by *his-3* targeting. Primers for *milR-1* promoter mutations are: mut1F, CGGGACGCACTACGGTTTAgACCTTCCCCCC, mut1R, GGGGGGAAGGTcTAAACCGTAGTGCGTCCCG, mut2F, GGTCCATGGTTCATgAACCCAAAATCCATTCGC, and mut2R, GCGAATGGATTTTGGGTTcATGAACCATGGACC.

The *dcl^dko^* strain was generated previously [37]. Liquid medium of 1× Vogel's, 0.1% glucose, and 0.17% arginine cultures with or without QA (0.01 M, pH 5.8), was used for liquid cultures. Race tube tests were carried out as previously described [34].

### Chromatin immunoprecipitation assay

Anti-c-Myc antibody was used to perform immunoprecipitation. The WT extract was used as control. qPCR was carried out to quantify the immunoprecipitated DNA. The relative DNA binding levels of ChIP assays were determined by comparing the relative enrichment of the ChIPPed DNA between the Myc-RPC7/RPB6 strain and a wild-type strain (lacking the Myc-tagged protein). rDNA levels in the ChIPPed DNA was used to normalize the loading in different samples.

### RNA polymerase III inhibitor treatment

ML-60218 (Calbiochem, catalog no. 557403) was used as an inhibitor of RNA polymerase III transcription [25]. ML-60218 was dissolved in DMSO at a concentration of 20 mg/ml. The final concentration used in tissue treatment was 20 mg/L. Culture medium containing an equal volume of DMSO or medium without DMSO was used as controls.

Conidia were germinated and grown in petri dishes containing liquid medium for one and a half days until even mycelia mats were formed. Discs were cut from the mats using a 7-mm cork borer and were shaken for 4 hrs in liquid medium before the addition of ML-60218. Tissue was treated at room temperature for 2 hours in the dark (to prevent light degradation of the inhibitor) before harvest.

### Northern blot, RT–PCR, and qPCR assays

Total RNA and small RNA was prepared as previously described [37], [38]. Small RNA was enriched from total RNA using 5% polyethylene glycol (MW8000) and 500 mM NaCl [39]. RNA concentration was measured by NanoDrop (Thermo Scientific NanoDrop ND-2000 1-position Spectrophotometer). Small RNA was separated by electrophoresis through 16% polyacrylamide, 7 M urea, 0.5× tris-borate EDTA (TBE) gels. Equal amounts of small RNA (20 µg) were loaded in each lane. Chemical cross-linking was used to fix small RNAs to Hybond-NX membrane (GE Healthcare). For siRNA hybridization, a single-stranded RNA was transcribed from a DNA template in antisense orientation in the presence of ^32^P-labeled uridine triphosphate (PerkinElmer) using MAXIscript T7 kit (Ambion). Then the labeled RNA was treated with TURBO DNase (Ambion) and hydrolyzed at 60°C for 2–3 hr to an average size of 50 nt probe using fresh alkalic solution (80 mM sodium bicarbonate and 120 mM sodium carbonate). The hydrolyzed probe was then added to ULTRAhyb buffer (Ambion) to perform hybridization at 42°C overnight. After hybridization, the membrane was washed three times with 2× SSC and 0.1% SDS buffer for 15 min at 42°C before it was exposed to X-ray film. For miRNA hybridization, probe was labeled with IDT DNA StarFire miRNA Detection Kit in the presence of ^32^P-labeled deoxyadenosine triphosphate (PerkinElmer). ULTRAhyb-Oligo Buffer (Ambion) was used for hybridization. Other conditions are the same with siRNA hybridization.

qRT-PCR was as previously described [37]. Reverse transcription of total RNA was carried out by using SuperScript II Reverse Transcriptase (RT) (Invitrogen) with random primers. qPCR of the cDNA was performed using iTaq SYBR Green Supermix with ROX (BIO-RAD). The qPCR primer sequences are listed in Table 1. The primer sequences used to verify the *pri-milR4* transcript were F1, 5′CTCAGTTGGTAGAGCGTTCG-3′, and R1, 5′-GAGGACCCATCTCACCAGAACA-3′.

**Table 1:** qPCR primer sequences.

gene	Primer F	Primer R
*rpb5*	TCAACAGTTCTGGTCGGCTGATGA	ATACGGATGGCTTTCAGCATTGCC
*rpc5*	TTATGGGAGTTGAGCCGAGTGCAA	ACCATCCAGCCACCGTCTGAAATA
*milR-1*	CTCTCCACTCCTTTATGTCC	CACTCCTCCACAGGGCACTT
*milR-2*	CTCAATCCCTACAGGTAGGT	CGATAGGAGAGTTGAACTCC
*milR-3*	GAAAGGCTGGAGACGTTTGC	GTTCACCATTGCTCTGAACG
*milR-4*	CCTTTCGCATACACCTAGGTAAGAA	CTCAGTTGGTAGAGCGTTCG
*milR-5*	TCATCACGAGAGCCCCATTTG	CTACCAGCAGCGAGCCAGACA
*milR-6*	CCGTGTTGCTTCGTTTTCAT	CCTAAGGGGTTGGTTCTCG
*milR-7*	AGGTCCCCGGATTCGTTC	AAAGCACCGCACCCAAGAT
*milR-8*	CCGGGTTGCTGTTAGATAGAA	ATACAATGCAAAACCGTCAC
*milR-9*	CAAAATACCCAGCTTCATCAT	CCAGCCGTACATTTATTCTTG
*milR-10*	CCGCTGCAGCTGAGTGTTC	GCTGTGCTAGGGGGAGTTGA
*rDNA*	CATTTCAACCATCAAGCTCTGC	CGACCATAGCGATGTAGAGTTAC
*tRNA*(*tRNA^Glu^*)	GGGTATGGTGTAGCGGTAACATC	GGGCTGTTGAGAGAGCAGTCA
*β-tubulin*	CTCCTCCTCCTCGTCAACACCA	CTCAAGATGTCCTCCACCTTCG

### Strand-specific poly(A) mRNA sequencing

Three independent wild-type cultures grown in liquid media were pooled for RNA isolation for sequencing. RNA sequencing and data analyses were performed by Beijing Genomic Institute in Shenzhen, China. Strand specific sequencing was carried out following the method introduced by Dmitri Parkhomchuk [40]. mRNAs that purified from total RNA were sheared and reverse-transcribed into first-strand cDNAs. dTTP was substituted by dUTP during the synthesis of the second-strand cDNAs. After cDNA fragmentation and adaptor ligation, the dUTP containing second strand population were removed by Uracil-N-Glycosylase. The resulting first strand cDNA population were sequenced by Illumina HiSeq 2000. The cleaned reads were assembled and mapped to the reference *Neurospora* genome sequence (http://www.broadinstitute.org/annotation/genome/neurospora/GenomeDescriptions.html#Neurospora_crassa_OR74A) using SOAP (Short Oligonucleotide Alignment Program) [41], tophat [42], [43] and cufflinks [44]–[46]. The strand-specific results were displayed and analyzed by IGV (Integrative Genomics Viewer) software [47].
